# Supplementary material for: Vitamin D Modified DSS-Induced Colitis in Mice via STING Signaling Pathway
Source: Biology (Basel). 2025 Jun 18;14(6):715. doi: 10.3390/biology14060715 (PMC12190092; doi:10.3390/biology14060715)
Supplement: Supplementary file 1 [file biology-14-00715-s001.zip › Supplementary Materials S1: 16s-report of gut microbiota/Groups/taxanomyBar/taxon_hist/without_unknown/top10/Class.distribution.pdf]

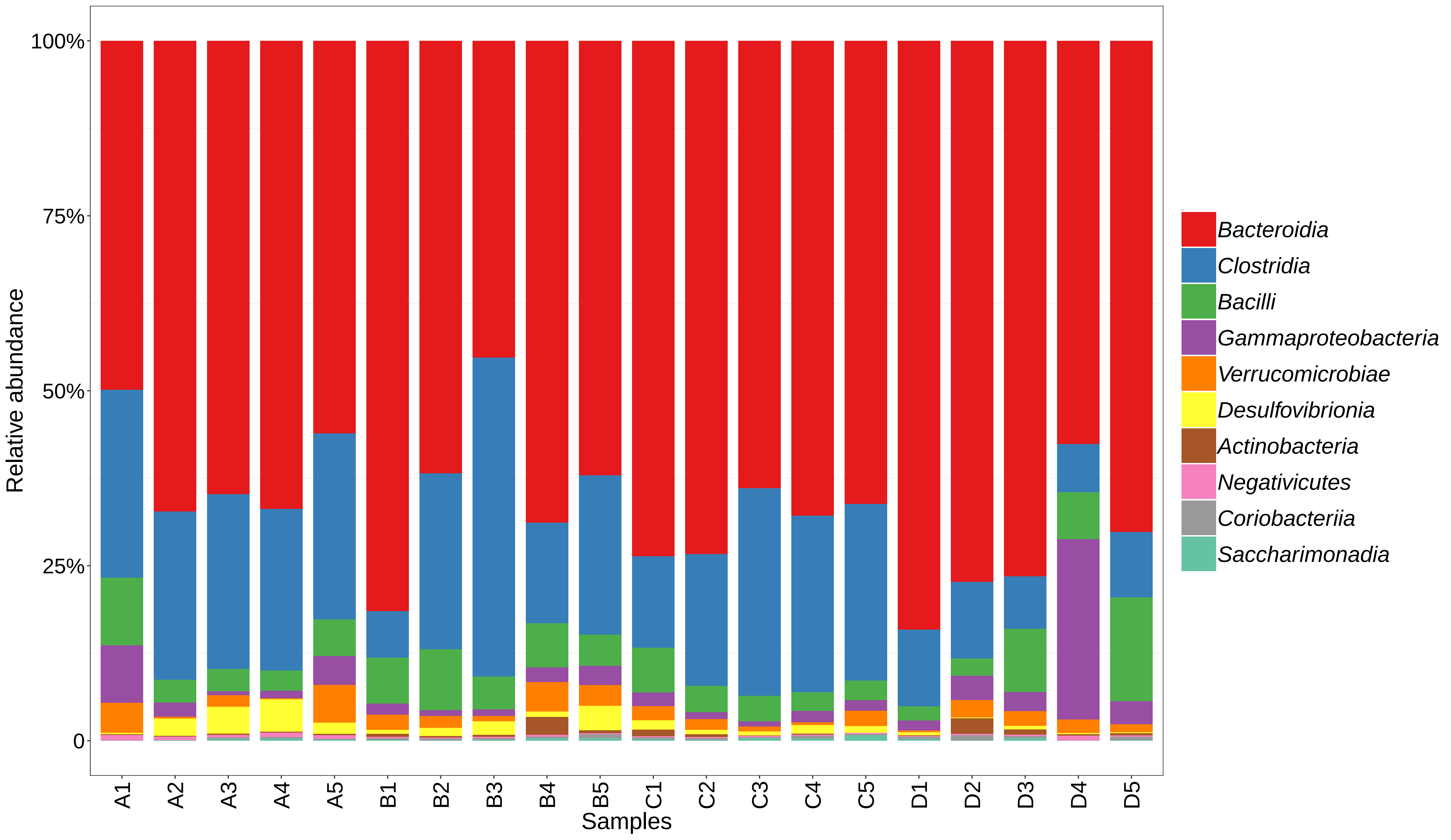
100%
75%
50%
25%
0
Relative abundance
A1
A2
A3
A4
A5
B1
B2
B3
B4
B5
C1
C2
C3
C4
C5
D1
D2
D3
D4
D5
Samples
Bacteroidia
Clostridia
Bacilli
Gammaproteobacteria
Verrucomicrobiae
Desulfovibrionia
Actinobacteria
Negativicutes
Coriobacteriia
Saccharimonadia
